# New species of *Yamadazyma* from rotting wood in China

**DOI:** 10.3897/mycokeys.83.71156

**Published:** 2021-08-26

**Authors:** Wan-Li Gao, Ying Li, Chun-Yue Chai, Zhen-Li Yan, Feng-Li Hui

**Affiliations:** 1 School of Life Science and Technology, Nanyang Normal University, Nanyang 473061, China Nanyang Normal University Nanyang China; 2 State Key Laboratory of Motor Vehicle Biofuel Technology, Henan Tianguan Enterprise Group Co., Ltd., Nanyang 473000, China State Key Laboratory of Motor Vehicle Biofuel Technology, Henan Tianguan Enterprise Group Co., Ltd Nanyang China

**Keywords:** Debaryomycetaceae, phylogeny, rotting wood-inhabiting yeast, taxonomy, *
Yamadazyma
*

## Abstract

*Yamadazyma* is one of the largest genera in the family Debaryomycetaceae (Saccharomycetales, Saccharomycetes) with species mainly found in rotting wood, insects and their resulting frass, but also recovered from flowers, leaves, fruits, tree bark, mushrooms, sea water, minerals, and the atmosphere. In the present study, several strains obtained from rotting wood in Henan and Yunnan Provinces of China were isolated. Based on morphology and a molecular phylogeny of the rDNA internal transcribed spacer region (ITS) and the D1/D2 domain of the large subunit (LSU) rDNA, these strains were identified as three new species: *Yamadazymaluoyangensis*, *Y.ovata* and *Y.paraaseri*; and three previously described species, *Y.insectorum*, *Y.akitaensis*, and *Y.olivae*. The three new species are illustrated and their morphology and phylogenetic relationships with other *Yamadazyma* species are discussed. Our results indicate a high undiscovered diversity of *Yamadazyma* spp. inhabiting rotting wood in China.

## Introduction

The genus *Yamadazyma*Billon-Grand (1989) was erected to accommodate 16 species previously assigned to the genus *Pichia* (Billon-Grand 1989). These species have coenzyme Q-9 as their main ubiquinone, form hat-shaped ascospores, produce pseudohyphae, ferment sugars, and require an exogenous source of vitamins for growth (Billon-Grand 1989; Kurtzman 2011). However, *Yamadazyma* was not initially accepted as genus due to a phylogenetic analysis of D1/D2 LSU rDNA strongly suggesting its polyphyletic nature (Kurtzman and Robnett 1998). Kurtzman and Suzuki (2010) analyzed phylogenetic relationships among species of *Pichia* and related genera based on combined sequences of the D1/D2 LSU rDNA and SSU rDNA, and proposed a new circumscription for *Yamadazyma* with six sexual species and 11 asexual species assigned to the genus *Candida* (Kurtzman and Suzuki 2010). *Yamadazyma* was later resolved as a well-supported monophyletic clade and a generally accepted genus in the family Debaryomycetaceae, order Saccharomycetales (Kurtzman and Suzuki 2010; Kurtzman 2011). The monophyly of the *Yamadazyma* clade was also supported by combined analysis of the ITS and D1/D2 LSU rDNA (Groenewald et al. 2011; Haase et al. 2017). In the fifth edition of The Yeasts: A Taxonomic Study, *Yamadazymaphilogaea*, the type species of the genus, as well as *Y.akitaensis*, *Y.mexicana*, *Y.nakazawae*, *Y.scolyti*, *Y.triangularis*, and 23 *Candida* species were placed in the *Yamadazyma* clade (Kurtzman 2011; Lachance et al. 2011). Since then, a few novel *Candida* species have been described from this clade, including *C.kanchanaburiensis* (Nakase et al. 2008), *C.khao-thaluensis*, *C.tallmaniae*, *C.oceani* (Burgaud et al. 2011), and *C.vaughaniae* (Groenewald et al. 2011). In addition, many new species, e.g., *Y.phyllophila*, *Y.paraphyllophila*, *Y.siamensis* (Kaewwichian et al. 2013), *Y.terventina* (Ciafardini et al. 2013), *Y.ubonensis* (Junyapate et al. 2014), *Y.dushanensis* (Wang et al. 2015), *Y.epiphylla*, *Y.insecticola* (Jindamorakot et al. 2015), *Y.riverae* (Lopes et al. 2015), *Y.barbieri* (Burgaud et al. 2016), *Y.endophytica* (Khunnamwong and Limtong 2016), *Y.kitorensis* (Nagatsuka et al. 2016), *Y.laniorum* (Haase et al. 2017), and *Y.cocois* (Maksimova et al. 2020), have been proposed as part of the genus, and three have already been transferred to *Yamadazyma* as new combinations: *Y.olivae*, *Y.tumulicola*, and *Y.takamatsuzukensis* (Nagatsuka et al. 2016). The *Yamadazyma* clade currently consists of 24 species of the genus *Yamadazyma* and 38 asexual species still assigned to the genus *Candida*, making it one of the largest genera tentatively assigned to the family Debaryomycetaceae (Groenewald et al. 2011; Kurtzman 2011; Maksimova et al. 2020). Among 24 species included in this genus, 7 were sexual morphs, viz. *Y.akitaensis*, *Y.mexicana*, *Y.nakazawae*, *Y.philogaea*, *Y.riverae*, *Y.scolyti*, and *Y.triangularis* (Kurtzman 2011; Lopes et al. 2015).

*Yamadazyma* species can be originally found in tropical, subtropical, and temperate regions of different continents, but most known species appear to exist in Asia and South America (Nakase et al. 2008; Groenewald et al. 2011; Kurtzman 2011; Lachance et al. 2011; Kaewwichian et al. 2013; Junyapate et al. 2014; Jindamorakot et al. 2015; Lopes et al. 2015; Wang et al. 2015; Burgaud et al. 2016; Khunnamwong and Limtong 2016; Nagatsuka et al. 2016). The genus has been heavily studied in Asia, and 17 species of *Yamadazyma* were previously reported in Japan and Thailand (Nakase et al. 2008; Groenewald et al. 2011; Kurtzman 2011; Lachance et al. 2011; Kaewwichian et al. 2013; Junyapate et al. 2014; Jindamorakot et al. 2015; Wang et al. 2015; Khunnamwong and Limtong 2016; Nagatsuka et al. 2016). By contrast, little is known about *Yamadazyma* spp. in China. To date, only three *Yamadazyma* species have been described in China, namely *C.diospyri*, *Y.dushanensis*, and *Y.paraphyllophila* (Lachance et al. 2011; Kaewwichian et al. 2013; Wang et al. 2015). In this study, we collected rotting wood samples from Yunnan and Henan Provinces in China. After isolation and examination, three new species and three known species of *Yamadazyma* were identified based on phenotypic characteristics and phylogenetic analysis, increasing the species diversity of *Yamadazyma* in China.

## Materials and methods

### Sample collection and yeast isolation

Samples of rotting wood were collected in the Xishuangbanna Primeval Forest Park (Yunnan Province, China) and the Tianchi Mountain National Forest Park (Henan Province, China). The Xishuangbanna Primeval Forest Park (21°98'N, 100°88'E) is 500 m above sea level (MASL), with a hot and humid climate. The average annual temperature is between 16 °C and 28 °C, and the average annual rainfall is above 1,100 mm. The Tianchi Mountain National Forest Park (34°33'N, 112°28'E) is at 850 MASL, with a continental monsoon climate, average annual temperature of 14–16 °C, and average annual rainfall between 800 mm and 900 mm. Fifty decayed wood samples were collected during July and August in 2018–2020. The samples were stored in sterile plastic bags and transported under refrigeration to the laboratory over a period of no more than 24 h. The yeast strains were isolated from rotting wood samples in accordance with the methods described by Wang et al. (2015). Each sample (1 g) was added to 20 ml sterile yeast extract-malt extract (YM) broth (0.3% yeast extract, 0.3% malt extract, 0.5% peptone, 1% glucose, pH 5.0 ± 0.2) supplemented with 0.025% sodium propionate and 200 mg/L chloramphenicol in a 150 ml Erlenmeyer flask and then cultured for 3–10 days on a rotary shaker. Subsequently, 0.1 ml aliquots of the enrichment culture and appropriate decimal dilutions were spread on YM agar plates and then incubated at 25 °C for 3–4 days. Different yeast colony morphotypes were then isolated by repeated plating on YM agar and then stored on YM agar slants at 4 °C or in 15% glycerol at – 80 °C.

### Phenotypic study

Morphological and physiological properties were determined according to those used by Kurtzman et al. (2011). The beginning of the sexual stage was determined by incubating single or mixed cultures of each of the two strains on cornmeal (CM) agar, 5% malt extract (ME) agar, dilute (1:9) V8 agar, or yeast carbon base plus 0.01% ammonium sulfate (YCBAS) agar at 15 and 25 °C for 6 weeks (Kurtzman 2011; Wang et al. 2015). The assimilation of carbon and nitrogen compounds and related growth requirements were tested at 25 °C. The effects of temperature from 25–40 °C were examined in liquid and agar plate cultures. Photomicrographs were taken using a Leica DM 2500 microscope (Leica Microsystems GmbH, Wetzlar, Germany) with a Leica DFC295 digital microscope color camera, with bright field, phase contrast, and DIC optics. Novel taxonomic descriptions and proposed names were deposited in MycoBank (http://www.mycobank.org; 8 June 2021) (Crous et al. 2004).

### DNA extraction, PCR amplification, and sequencing

Genomic DNA was extracted from the yeast using an Ezup Column Yeast Genomic DNA Purification Kit, according to the manufacturer’s instructions (Sangon Biotech, Shanghai, China). The internal transcribed spacer (ITS) and the D1/D2 domain of the large subunit (LSU) rDNA were respectively amplified using ITS5/ITS4 (White et al. 1990) and NL1/NL4 (Kurtzman and Robnett 1998) primers with cycling conditions of 94 °C/30 s, 55 °C/50 s, 72 °C/60 s. All the PCR protocols had 35 cycles including 94 °C/5 min initial denaturation and 72 °C/10 min final extension.

The 25 µL total volume of PCR mixture contained 9.5 µL of ddH2O, 12.5 µL of 2X PCR Master Mix (TIANGEN Co., China), 1 µL of DNA template, and 1 µL of forward and reverse primers (10 µM each) in each reaction. PCR amplified products were checked on 1% agarose electrophoresis gels stained with GoldView I nuclear staining dye (1 µL/10 mL of agarose). Purification and sequencing of PCR products were done by Sangon Biotech (Shanghai) Co., Ltd., Shanghai, China. A consensus sequence for each gene region was assembled in SeqMan (DNAStar, Inc., Madison, WI, USA). The newly-generated sequences were deposited in GenBank (https://www.ncbi.nlm.nih.gov/genbank/ (accessed on 30 May 2021); Table 1).

Abbreviations:

**CBS**CBS-KNAW Collections, Westerdijk Fungal Biodiversity Institute, Utrecht, The Netherlands;

**CECT**the Spanish Type Culture Collection, Valencia, Spain;

**VTCC**Vietnam Type Culture Collection, Hanoi, Vietnam;

**NYNU** Microbiology Lab, Nanyang Normal University, Henan, China;

**T** type strain.

### Phylogenetic analysis

The sequences obtained in this study and the reference sequences downloaded from GenBank (Table 1) were aligned using MAFFT v 7(https://mafft.cbrc.jp/alignment/server/large.html;) (Katoh et al. 2019) and manually edited using MEGA7 (Kumar et al. 2016). The best-fit nucleotide substitution models for individual and combined datasets were selected using jModelTest v2.1.7 (Darriba et al. 2012) according to the Akaike information criterion. Phylogenetic analyses of combined gene regions (ITS and D1/D2 LSU) were performed using MEGA7 for maximum parsimony (MP) analysis (Kumar et al. 2016) and PhyML v3.0 for maximum likelihood (ML) analysis (Guindon et al. 2010). *Scheffersomycescoipomoensis* (CBS 8178) and *Babjeviellainositovora* (CBS 8006) were used as the outgroup taxa based on Haase et al. (2017) and Nagatsuka et al. (2016).

**Table 1.** Sequences used in molecular phylogenetic analysis. Entries in bold are newly generated in this study.

Species	Strain no.	Locality	Sample	GenBank accession numbers	
				ITS	D1/D2
* Candida aaseri *	CBS 1913^T^	Norway	Sputum	AY821838	U45802
* C. amphixiae *	CBS 9877^T^	Panama	Beetle	EU491501	AY520327
* C. andamanensis *	CBS 10859^T^	Thailand	Estuarine water	AB525239	AB334210
* C. atlantica *	CBS 5263^T^	Portugal	Shrimp egg	AJ539368	U45799
* C. atmosphaerica *	CBS 4547^T^	Spain	Atmosphere	AJ539369	U45779
* C. blattariae *	CBS 9876^T^	Panama	Cockroach	FJ715435	AY640213
* C. buinensis *	CBS 6796^T^	Papua New Guinea	Gelatinous exudate	HQ283376	U45778
* C. cerambycidarum *	CBS 9879^T^	Panama	Beetle	AY964669	AY520299
* C. conglobata *	CBS 2018^T^	–	Tubercular lung	AJ539370	U45789
* C. dendronema *	CBS 6270^T^	South Africa	Frass	HQ283365	U45751
* C. diddensiae *	CBS 2214^T^	USA	Shrimp	AY580315	U45750
* C. diospyri *	CBS 9769^T^	China	Kaki fruit	AY450919	AY450918
* C. endomychidarum *	CBS 9881^T^	Panama	Beetle	AY964672	AY520330
* C. friedrichii *	CBS 4114^T^	Germany	D-glucitol solution	HQ283377	U45781
* C. germanica *	CBS 4105^T^	Germany	Atmosphere	HQ283366	AF245401
* C. gorgasii *	CBS 9880^T^	Panama	Beetle	AY964670	AY520300
* C. insectorum *	CBS 6213^T^	South Africa	Frass	HQ283372	U45791
*** C. insectorum ***	**NYNU 1672**	**China**	**Rotten wood**	**MZ314279**	**MZ314278**
* C. jaroonii *	CBS 10790^T^	Thailand	Frass	AB360437	DQ404493
* C. kanchanaburiensis *	CBS 11266^T^	Thailand	Mushroom	NR_137581	KY106534
* C. keroseneae *	CECT 13058^T^	UK	Aircraft fuel	FJ235128	FJ357698
* C. khao-thaluensis *	CBS 8535^T^	Thailand	Leaf	HQ283374	HQ283383
* C. koratica *	CBS 10789^T^	Thailand	Frass	AB360443	AB354232
* C. lessepsii *	CBS 9941^T^	Panama	Beetle	AY964671	AY640214
* C. membranifaciens *	CBS 1952^T^	India	Urine	AJ606465	U45792
* C. michaelii *	CBS 9878^T^	Panama	Beetle	AY964673	AY520329
* C. naeodendra *	CBS 6032^T^	South Africa	Frass	AY580316	U45759
* C. oceani *	CBS 11857^T^	Atlantic Ocean	Deep-sea coral	NR_156008	GU002284
* C. pseudoaaseri *	CBS 11170^T^	Germany	Blood culture	JN241686	JN241689
* C. sinolaborantium *	CBS 9940^T^	Panama	Beetle	NR_111343	NG_055206
* C. songkhlaensis *	CBS 10791^T^	Thailand	Frass	AB360438	DQ404499
* C. spencermartinsiae *	CBS 10894^T^	Seawater	Florida	FJ008050	FJ008044
* C. tallmaniae *	CBS 8575^T^	French Guiana	Flower	HQ283378	HQ283385
* C. tammaniensis *	CBS 8504^T^	USA	Frass	HQ283375	AF017243
* C. taylori *	CBS 8508^T^	Belize	Sea water	FJ008051	FJ008045
* C. temnochilae *	CBS 9938^T^	Panama	Beetle	AY964678	AY242344
* C. trypodendroni *	CBS 8505^T^	Canada	Beetle	FJ153212	AF017240
* C. vaughaniae *	CBS 8583^T^	French Guiana	Flower	HQ283364	HQ283381
* C. vrieseae *	CBS 10829^T^	Brazil	Bromeliad	FJ755905	EU200785
* Yamadazyma akitaensis *	CBS 6701^T^	Japan	Exudate	DQ409164	U45766
*** Y. akitaensis ***	**NYNU 16719**	**China**	**Rotten wood**	**MZ314281**	**MZ314280**
* Y. barbieri *	CBS 14301^T^	Brazil	Sea water	LT547714	LT547716
* Y. cocois *	VTCC 920004^T^	Vietnam	Fruits of the coconut palm	MN764369	MN764369
* Y. dushanensis *	CBS 13914^T^	China	Rotten wood	KM272249	KM272248
* Y. endophytica *	CBS 14163^T^	Thailand	Corn leaf	KT307981	KT307981
* Y. epiphylla *	CBS 13384^T^	Thailand	Rice leaf	LC006082	LC006026
* Y. insecticola *	CBS 13382^T^	Thailand	Frass	LC006081	DQ400379
* Y. kitorensis *	CBS 14158^T^	Japan	Red viscous gel	LC060995	LC060995
* Y. laniorum *	CBS 14780^T^	USA	Bark	KY588337	KY588136
*** Y. luoyangensis ***	**NYNU 201023^T^**	**China**	**Rotting wood**	**MW365549**	**MW365545**
*** Y. luoyangensis ***	**NYNU 201035**	**China**	**Rotting wood**	**MZ318445**	**MZ318422**
* Y. mexicana *	CBS 7066^T^	Mexico	Agria cactus	AB054110	U45797
* Y. nakazawae *	CBS 6700^T^	Japan	Exudate	EU343867	U45748
* Y. olivae *	CBS 11171^T^	Greece	Fermenting olive	FJ715432	FJ715430
*** Y. olivae ***	**NYNU 167106**	**China**	**Rotting wood**	**MZ314288**	**MZ314282**
*** Y. olivae ***	**NYNU 209116**	**China**	**Rotting wood**	**MZ318443**	**MZ318444**
*** Y. ovata ***	**NYUN 191125^T^**	**China**	**Rotting wood**	**MT990560**	**MT990559**
*** Y. ovata ***	**NYUN 19130**	**China**	**Rotting wood**	**MZ318424**	**MZ318425**
*** Y. ovata ***	**NYUN 19116**	**China**	**Rotting wood**	**MZ318442**	**MZ318423**
*** Y. paraaseri ***	**NYNU 1811114^T^**	**China**	**Rotting wood**	**MK682794**	**MK682805**
*** Y. paraaseri ***	**NYNU 181033**	**China**	**Rotting wood**	**MZ318421**	**MZ318460**
* Y. paraphyllophila *	CBS 9928^T^	China, Taiwan	Pencil wood leaf	AY559447	AY562397
* Y. philogaea *	CBS 6696^T^	South Africa	Soil	AB054107	U45765
* Y. phyllophila *	CBS 12572^T^	Thailand	Corn leaf	AB734050	AB734047
* Y. riverae *	CBS 14121^T^	Brazil	Rotting wood	KP900044	KP900043
* Y. scolyti *	CBS 4802^T^	USA	Frass	EU343807	U45788
* Y. siamensis *	CBS 12573^T^	Thailand	Sugarcane leaf	AB734049	AB734046
* Y. takamatsuzukensis *	CBS 10916^T^	Japan	Air	AB365470	AB365470
* Y. tenuis *	CBS 615^T^	Russia	Beetle	HQ283371	U45774
* Y. terventina *	CBS 12510^T^	Italy	Olive oil	JQ247717	JQ247717
* Y. triangularis *	CBS 4094^T^	Japan	Tamari soya	EU343869	U45796
* Y. tumulicola *	CBS 10917^T^	Japan	Stone chamber	AB365463	AB365463
* Y. ubonensis *	CBS 12859^T^	Thailand	Tree bark	NR_155998	AB759913
* Scheffersomyces coipomoensis *	CBS 8178^T^	–	–	NR_111424	U45747
* Babjeviella inositovora *	CBS 8006^T^	–	–	NR_111018	U45848

MP analysis was run using a heuristic search option of 1,000 search replicates with random-addition of sequences and tree bisection and reconnection (TBR) as the branch-swapping algorithm. Gaps were treated as missing data. Bootstrapping with 1,000 replicates was performed to determine branch support (Felsenstein 1985). Parsimony scores of tree length (TL), consistency index (CI), retention index (RI), and rescaled consistency (RC) were calculated for each generated tree. ML analysis was performed using a GTR site substitution model, including a gamma-distributed rate heterogeneity and a proportion of invariant sites (Guindon et al. 2010). Branch support was evaluated using a bootstrapping method of 1,000 bootstrap replicates (Hillis and Bull 1993). The phylogenies from MP and ML analyses were displayed using Mega7 and FigTree v1.4.3 (Rambaut 2016), respectively. ML and MP bootstrap support values above 50% are shown as first and second positionS above nodes, respectively.

## Results

### Molecular phylogeny

**Figure 1.**
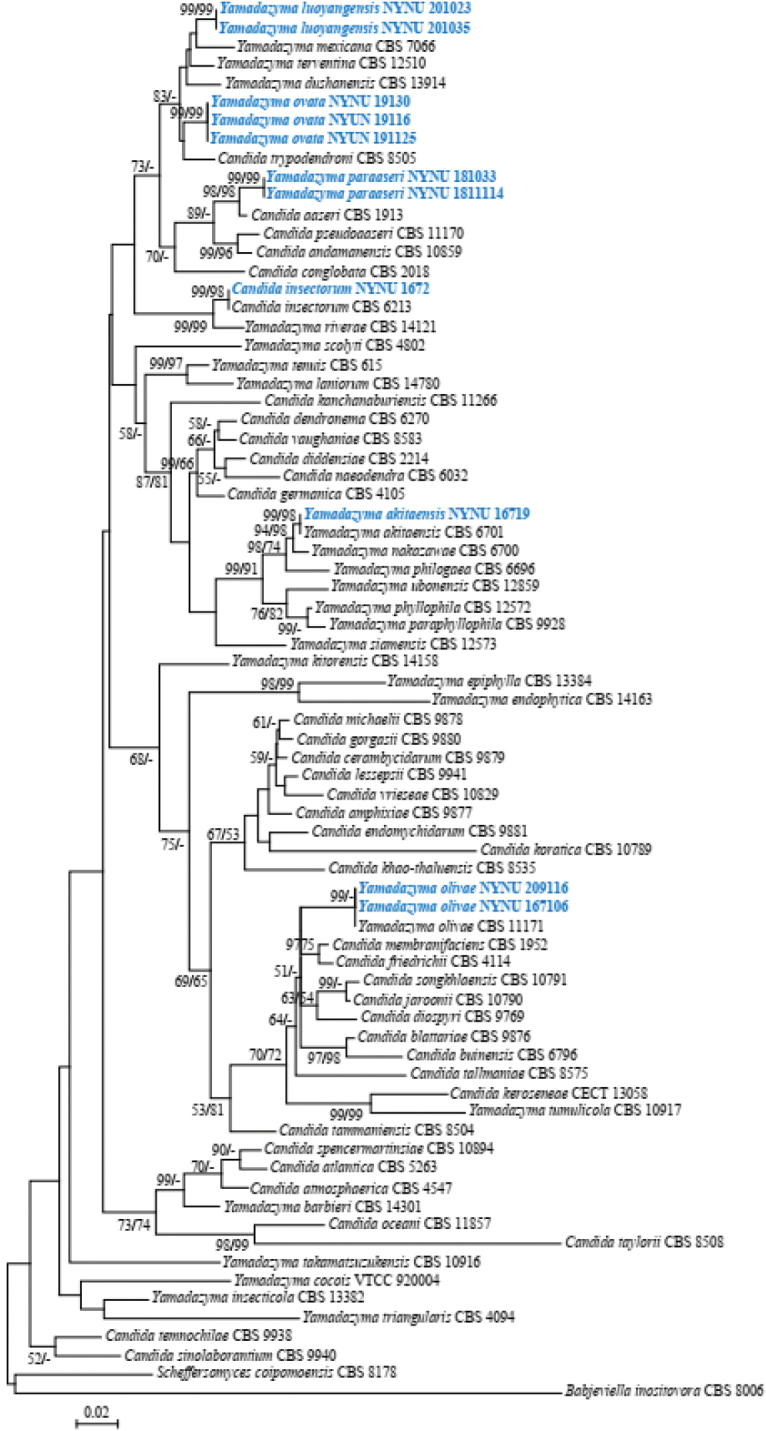
Maximum likelihood phylogenetic tree of *Yamadazyma* inferred from the combined ITS and D1/D2 LSU dataset and rooted with *Scheffersomycescoipomoensis* (CBS 8178) and *Babjeviellainositovora* (CBS 8006). ML and MP bootstrap support values above 50% are respectively shown at the first and second positions. Newly sequenced collections are in blue boldface.

The alignment based on the combined nuclear dataset (ITS and D1/D2 LSU) included 65 taxa and two outgroup taxa (*Scheffersomycescoipomoensis* and *Babjeviellainositovora*), and was comprised of 1,103 characters including gaps (576 for ITS and 527 for D1/D2 LSU) in the aligned matrix. Of these characters, 351 were constant, 455 variable characters were parsimony-uninformative, and 297 characters were parsimony-informative. The heuristic search using MP analysis generated the most parsimonious tree (TL = 979, CI = 0.297, RI = 0.653, RC = 0.248). The best model applied in the ML analysis was GTR+I+G. The ML analysis yielded a best scoring tree with a final optimization likelihood value of –11,006.61. Both methods for phylogenetic tree inference resulted in a similar topology. Therefore, only the best scoring PhyML tree is shown with BS and BT values simultaneously in Figure 1.

According to the phylogenetic tree (Figure 1), three known species, *Y.insectorum*, *Y.akitaensis*, and *Y.olivae*, were part of *Yamadazyma*. *Yamadazymaluoyangensis*, *Y.ovata*, and *Y.paraaseri* are new to science based on the distinct and well-supported molecular phylogenetic placement and morphological differences with their closest described relatives (Table 2). Phylogenetically, *Y.luoyangensis* clustered together with *Y.ovata* and other species, including *Y.mexicana*, *Y.terventina*, *Y.dushanensis*, and *C.trypodendroni*, while *Y.paraaseri* was closely related to *C.aaseri* with high bootstrap support (98% ML/98% MP).

**Table 2.** Physiological characteristics of the new *Yamadazyma* species and their closely related taxa.

Characteristics	* Y. luoyangensis *	* Y. mexicana *	* Y. ovata *	* C. trypodendroni *	* Y. paraaseri *	* C. aaseri *
Fermentation of						
d-Glucose	+	+	+	+	-	v
Assimilation of						
l-Sorbose	–	–	+	–	+	s
d-Glucosamine	+	+	+	–	+	–
l-Rhamnose	+	+	–	+	–	–
Melibiose	–	v	+	–	–	–
Lactose	–	+	–	–	+	v
Raffinose	–	+	–	–	–	–
Inulin	+	–	–	–	+	–
Xylitol	+		+	–	–	+
Galactitol	+	v	+	–	–	–
2-Keto-d-Gluconate	–	s	+	+	–	–
Cadaverine	–	n	–	+	–	+
Growth tests						
10%Nacl/5%glucose	+	+	+	v	–	+
Growth at 37 °C	–	+	+	–	+	+

+, positive reaction; –, negative reaction; s, slow positive reaction; v, variable reaction; n, data not available.

### Taxonomy

#### Yamadazyma luoyangensis

Taxon classificationFungiSaccharomycetalesDebaryomycetaceae

C.Y. Chai & F.L. Hui sp. nov.

2ED16117-1D8F-5F51-AABF-55CF47C86EC4

840099

Figure 2

##### Type.

China, Henan Province, Luoyang City, Song County, in rotting wood from a forest park, September 2020, J.Z. Li & Z.T Zhang (holotype NYNU 201023^T^, culture ex-type CBS 16666, CICC 33509).

##### Etymology.

The species name *luoyangensis* refers to the geographical origin of the type strain of this species.

##### Description.

**Figure 2.**
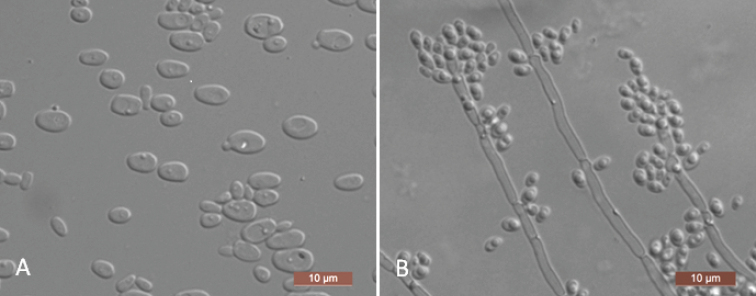
*Yamadazymaluoyangensis* (NYNU 201023, holotype) **A** budding cells after three days in YM broth at 25 °C **B** pseudohyphae on cornmeal agar after seven days at 25 °C. Scale bars: 10 μm.

The cells are ovoid to ellipsoid (2–4 × 3.5–7 μm) and occur singly or in pairs after being placed in YM broth for three days at 25 °C (Figure 2A). Budding is multilateral. After three days of growth on YM agar at 25 °C, the colonies are white to cream-colored, buttery, and smooth, with entire margins. After seven days at 25 °C on a Dalmau plate culture with CM agar, pseudohyphae are formed, but true hyphae are not (Figure 2B). Asci or signs of conjugation are not observed on sporulation media. Glucose, galactose, trehalose, and cellobiose are fermented, but maltose, sucrose, melibiose, lactose, melezitose, raffinose, d-xylose, and inulin are not. Glucose, galactose, d-glucosamine, d-ribose, d-xylose, l-arabinose, d-arabinose, l-rhamnose, sucrose, maltose, trehalose, methyl α-d-glucoside, cellobiose, salicin, arbutin, melezitose, inulin, glycerol, erythritol, ribitol, d-glucitol, d-mannitol, galactitol, d-glucono-1, 5-lactone, 5-keto-d-gluconate, d-gluconate, succinate, citrate, and ethanol are assimilated. No growth is observed in l-sorbose, melibiose, lactose, raffinose, *myo*-inositol, 2-keto-d-gluconate, d-glucuronate, dl-lactate, or methanol. In nitrogen-assimilation tests, growth is present on ethylamine, l-lysine, glucosamine, and d-tryptophan, while growth is absent on nitrate, nitrite, cadaverine, creatine, creatinine, and imidazole. Growth is observed at 35 °C but not at 37 °C. Growth in the presence of 10% NaCl with 5% glucose is present, but growth in the presence of 0.01% cycloheximide and 1% acetic acid is absent. Starch-like compounds are not produced. Urease activity and diazonium blue B reactions are negative.

##### Additional isolate examined.

China, Henan Province, Luoyang City, Song County, in rotting wood from a forest park, September 2020, J.Z. Li & Z.T Zhang, NYNU 201035.

##### GenBank accession numbers.

Holotype NYNU 201023^T^ (ITS: MW365549; D1/D2 LSU: MW365545); additional isolate NYNU 201035 (ITS: MZ318445; D1/D2 LSU: MZ318422).

##### Notes.

Two isolates representing *Y.luoyangensis* were resolved in a well-supported clade and are most closely related to *Y.mexicana* (Figure 1). *Yamadazymaluoyangensis* can be distinguished from *Y.mexicana* based on ITS and D1/D2 LSU loci (4/592 in ITS and 10/531 in D1/D2 LSU). Physiologically, *Y.luoyangensis* differs from *Y.mexicana* by its ability to assimilate inulin and 5-keto-d-gluconate and its inability to assimilate lactose, raffinose, and 2-keto-d-gluconate. Additionally, *Y.mexicana* grows at 37 °C, while *Y.luoyangensis* does not (Table 2) (Kurtzman 2011).

#### Yamadazyma ovata

Taxon classificationFungiSaccharomycetalesDebaryomycetaceae

C.Y. Chai & F.L. Hui sp. nov.

D54BA6DF-9A19-5101-B54E-21CAC2344F6D

840100

Figure 3

##### Type.

China, Henan Province, Luoyang City, Song County, in rotting wood from a forest park, September 2019, J.Z. Li & Z.T Zhang (holotype NYNU 191125^T^, culture ex-type CBS 16655, CICC 33500).

##### Etymology.

The species name *ovata* refers to the ovoid cell morphology of the type strain.

##### Description.

**Figure 3.**
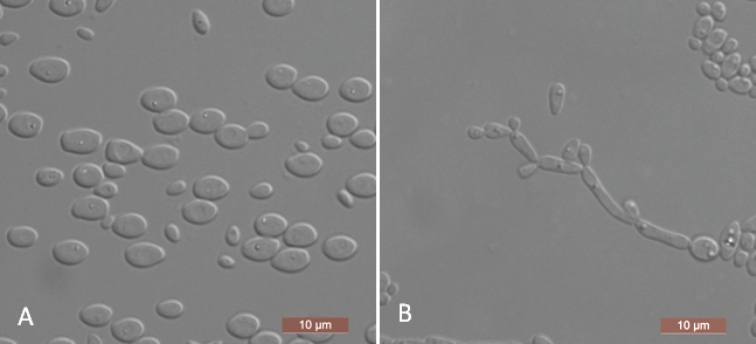
*Yamadazymaovata* (NYNU 191125, holotype) **A** budding cells after three days in YM broth at 25 °C **B** pseudohyphae on cornmeal agar after nine days at 25 °C. Scale bars: 10 μm.

The cells are ovoid to ellipsoid (2–3 × 3–6.5 μm) and occur singly or in pairs after growth in a YM broth for three days at 25 °C (Figure 3A). Budding is multilateral. After three days of growth on YM agar at 25 °C, the colonies are white to cream-colored, buttery, and smooth with entire margins. After nine days at 25 °C, on a Dalmau plate culture with CM agar, pseudohyphae consisting of elongated cells with lateral buds are formed (Figure 3B). True hyphae are not observed. Asci or signs of conjugation are not observed on sporulation media. Glucose, galactose, and trehalose are fermented, but maltose, sucrose, melibiose, lactose, cellobiose, melezitose, raffinose, d-xylose, and inulin are not. Glucose, galactose, l-sorbose, d-glucosamine, d-ribose, d-xylose, l-arabinose, d-arabinose, sucrose, maltose, trehalose, methyl α-d-glucoside, cellobiose, salicin, melibiose, melezitose, glycerol, erythritol, ribitol, xylitol, d-glucitol, d-mannitol, d- galactitol, d-glucono-1, 5-lactone, 2-keto-d-gluconate, d-gluconate, succinate, citrate, and ethanol are assimilated. No growth is observed in l-rhamnose, lactose, raffinose, inulin, *myo*-inositol, d-glucuronate, dl-lactate, or methanol. In nitrogen-assimilation tests, growth is present on l-lysine, creatine, glucosamine, and d-tryptophan, while growth is absent on nitrate, nitrite, ethylamine, cadaverine, creatinine, or imidazole. Growth is observed at 37 °C, but not at 40 °C. Growth in the presence of 16% NaCl with 5% glucose is present, but growth in the presence of 0.01% cycloheximide and 1% acetic acid is absent. Starch-like compounds are not produced. Urease activity and diazonium blue B reactions are negative.

##### Additional isolates examined.

China, Henan Province, Luoyang City, Song County, in rotting wood from a forest park, September 2019, J.Z. Li & Z.T Zhang, NYNU 19116, NYNU 19130.

##### GenBank accession numbers.

Holotype NYNU 191125^T^ (ITS: MT990560; D1/D2 LSU: MT990559); additional isolates NYNU 19116 (ITS: MZ318442; D1/D2 LSU: MZ318423), and NYNU 19130 (ITS: MZ318424; D1/D2 LSU: MZ318425).

##### Notes.

We generated sequences for three isolates of *Y.ovata*, NYNU 191125, NYNU 19116, and NYNU 19130. This new species is phylogenetically most closely related to *C.trypodendroni* (Figure 1). *Yamadazymaovata* can be distinguished from *C.trypodendroni* based on ITS and D1/D2 LSU loci (15/565 in ITS and 8/532 in D1/D2 LSU). Physiologically, *Y.ovata* can be differentiated from *C.trypodendroni* based on growth in l-sorbose, d-glucosamine, melibiose, and d-glucono-1, 5-lactone, all of which are positive for *Y.ovata* and negative for *C.trypodendroni* (Table 2) (Lachance et al. 2011).

#### Yamadazyma paraaseri

Taxon classificationFungiSaccharomycetalesDebaryomycetaceae

C.Y. Chai & F.L. Hui sp. nov.

C541BB74-74E8-579A-B274-C2FCACE95A25

840101

Figure 4

##### Type.

China, Yunnan Province, Jinghong City, Mengyang Town, in rotting wood from a tropical rainforest, July 2018, K.F. Liu & Z.W. Xi (holotype NYNU 1811114^T^, culture ex-type CBS 16010, CICC 33365).

##### Etymology.

The species name *paraaseri* refers to its phylogenetic similarity to *C.aaseri*.

##### Description.

**Figure 4.**
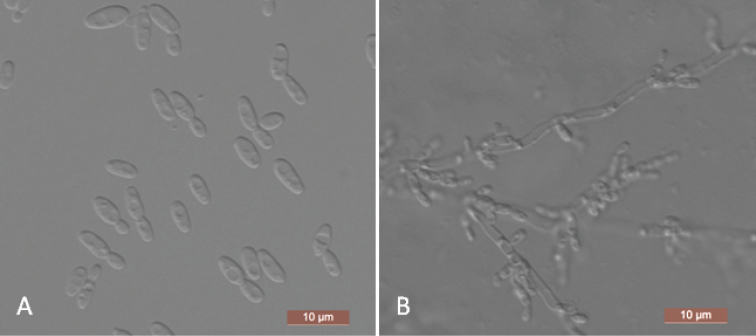
*Yamadazymaparaaseri* (NYNU 1811114, holotype) **A** budding cells after three days in YM broth at 25 °C **B** pseudohyphae on cornmeal agar after two weeks at 25 °C. Scale bars: 10 μm.

The cells are ovoid to elongate (2–2.5 × 3–8.5 μm) and occur singly or in pairs after being placed in YM broth for three days at 25 °C (Figure 4A). Budding is multilateral. After three days of growth on YM agar at 25 °C, the colonies are white to cream-colored, buttery, and smooth, with entire margins. After two weeks at 25 °C on a Dalmau plate culture with CM agar, pseudohyphae consisting of elongated cells with lateral buds are formed (Figure 4B). True hyphae are not observed. Asci or signs of conjugation are not observed on sporulation media. Fermentation of sugars is absent. Glucose, galactose, l-sorbose, d-glucosamine, d-ribose, d-xylose, l-arabinose, d-arabinose, sucrose, maltose, trehalose, methyl α-d-glucoside, cellobiose, salicin, arbutin, lactose, melezitose, inulin, glycerol, erythritol, ribitol, d-glucitol, d-mannitol, d-gluconate, dl-lactate, succinate, citrate, and ethanol are assimilated. No growth is observed in l-rhamnose, melibiose, raffinose, xylitol, galactitol, *myo*-inositol, d-glucono-1, 5-lactone, 2-keto-d-gluconate, d-glucuronate, or methanol. In nitrogen-assimilation tests, growth is present on ethylamine, l-lysine, glucosamine, and d-tryptophan, while growth is absent on nitrate, nitrite, cadaverine, creatine, creatinine, and imidazole. Growth is observed at 37 °C but not at 40 °C. Growth in the presence of 0.01% cycloheximide, 10% NaCl with 5% glucose and 1% acetic acid is absent. Starch-like compounds are not produced. Urease activity and diazonium blue B reactions are negative.

##### Additional isolate examined.

China, Yunnan Province, Jinghong City, Mengyang Town, in rotting wood from a tropical rainforest, July 2018, K.F. Liu & Z.W. Xi, NYNU 181033.

##### GenBank accession numbers.

Holotype NYNU 1811114^T^ (ITS: MK682794; D1/D2 LSU: MK682805); additional isolate NYNU 181033 (ITS: MZ318421; D1/D2 LSU: MZ318460).

##### Notes.

Two strains representing *Y.paraaseri* were clustered in a well-supported clade and were phylogenetically related to *C.aaseri* [7]. *Yamadazymaparaaseri* can be distinguished from *C.aaseri* based on ITS and D1/D2 LSU loci (8/573 in ITS and 8/531 in D1/D2 LSU). Physiologically, the ability to assimilate d-glucosamine and inulin and the inability to assimilate xylitol and d-glucono-1, 5-lactone are the primary differences between *Y.paraaseri* and its closest relative, *C.aaseri*. Additionally, *C.aaseri* can grow in 10% NaCl with 5% glucose, while *Y.paraaseri* cannot (Table 2) (Lachance et al. 2011).

## Discussion

In this work, six *Yamadazyma* species were identified based on morphology and molecular phylogeny. All species were isolated from rotting wood collected in Henan and Yunnan Provinces, China. *Yamadazymaluoyangensis*, *Y.ovata*, and *Y.paraaseri* are proposed as new species in *Yamadazyma* due to their well-supported phylogenic positions and distinctive physiological traits. Also, three known species of *Yamadazyma*, *Y.insectorum*, *Y.akitaensis*, and *Y.olivae*, were clearly identified by both morphological and molecular approaches.

In the past, methods of species identification of *Yamadazyma* were based only on morphology and physiological characters such as the shape of ascospores and reactions in standard growth and fermentation tests (Billon-Grand 1989). Recent molecular phylogenetic analyses demonstrate that determining species boundaries using only morphology and physiological characters is not possible due to their variability under changing environmental conditions (Kurtzman 2011; Lachance et al. 2011). D1/D2 LSU sequence is an appropriate marker to identify species of *Yamadazyma* species through phylogenetic analysis, as revealed by Kurtzman and Robnett (1998). Many *Yamadazyma* species are described based on a polyphasic approach together with morphological and physiological characterization (Suh et al. 2005; Kurtzman 2007; Imanishi et al. 2008; Nagatsuka et al. 2009; Am-In et al. 2011). However, none to only two substitutions are present in D1/D2 LSU sequences of the ex-type strains of the closest related species within *Yamadazyma*, such as *C.diddensiae* and *C.naeodendra*, *Y.akitaensis* and *Y.nakazawae* as well as *C.jaroonii* and *C.songkhlaensis* (Groenewald et al. 2011; Wang et al. 2015). The ITS sequences show more variation between these closely related well-defined species in contrast to the low nucleotide differences in D1/D2 LSU sequences (Groenewald et al. 2011). Although D1/D2 LSU sequence is still an appropriate region to use for higher level taxon delimitations, it is clear that this sequence alone is not sufficient for species delimitation in the *Yamadazyma* clade. The ITS sequence is thus a good additional marker to obtain a better understanding of relatedness among *Yamadazyma* species.

*Yamadazyma* species have a worldwide distribution and are isolated from diverse substrates. They can be found in flowers, leaves, fruits, tree bark, mushrooms, sea water, mineral and atmosphere, but most known species appear to exist in rotting wood, insects and their resulting frass (Groenewald et al. 2011; Kurtzman 2011). This clade also includes the clinically significant species *C.aaseri*, *C.conglobata*, *C.pseudoaaseri*, and *Y.triangularis* (Kurtzman 2011; Lachance et al. 2011). These studies expanded our knowledge on the substrates where *Yamadazyma* species can occur, but on the other hand demonstrated the complicated ecological function of this genus. In this study, three known species and three new species were identified from rotting wood in China. Further research will focus on *Yamadazyma* diversity from a wide range of substrates.

## Supplementary Material

XML Treatment for Yamadazyma luoyangensis

XML Treatment for Yamadazyma ovata

XML Treatment for Yamadazyma paraaseri
